# Pulsed laser deposited GeTe-rich GeTe-Sb_2_Te_3_ thin films

**DOI:** 10.1038/srep26552

**Published:** 2016-05-20

**Authors:** M. Bouška, S. Pechev, Q. Simon, R. Boidin, V. Nazabal, J. Gutwirth, E. Baudet, P. Němec

**Affiliations:** 1 Department of Graphic Arts and Photophysics, Faculty of Chemical Technology, University of Pardubice, Studentská 573, 53210 Pardubice, Czech Republic; 2 Institut de Chimie de la Matière Condensée de Bordeaux – CNRS, 87, av. du Dr. Albert Schweitzer, 33608 Pessac Cedex, France; 3 Equipe Verres et Céramiques, UMR-CNRS 6226, Sciences Chimiques de Rennes (SCR), Université de Rennes 1, 35042 Rennes Cedex, France

## Abstract

Pulsed laser deposition technique was used for the fabrication of Ge-Te rich GeTe-Sb_2_Te_3_ (Ge_6_Sb_2_Te_9_, Ge_8_Sb_2_Te_11_, Ge_10_Sb_2_Te_13_, and Ge_12_Sb_2_Te_15_) amorphous thin films. To evaluate the influence of GeTe content in the deposited films on physico-chemical properties of the GST materials, scanning electron microscopy with energy-dispersive X-ray analysis, X-ray diffraction and reflectometry, atomic force microscopy, Raman scattering spectroscopy, optical reflectivity, and sheet resistance temperature dependences as well as variable angle spectroscopic ellipsometry measurements were used to characterize as-deposited (amorphous) and annealed (crystalline) layers. Upon crystallization, optical functions and electrical resistance of the films change drastically, leading to large optical and electrical contrast between amorphous and crystalline phases. Large changes of optical/electrical properties are accompanied by the variations of thickness, density, and roughness of the films due to crystallization. Reflectivity contrast as high as ~0.21 at 405 nm was calculated for Ge_8_Sb_2_Te_11_, Ge_10_Sb_2_Te_13_, and Ge_12_Sb_2_Te_15_ layers.

During the last twenty years, the thin films from GeSbTe (hereinafter GST) or AgInSbTe system have been deeply investigated^1^,^2^,^3^,^4^,^5^,^6^,^7^. The main reason for high scientific interest in this class of materials is the fact that these materials are able to transform quickly and reversibly between amorphous and crystalline phases (disorder-order transition); this phenomenon was first reported by Ovshinsky^8^. Phase transition can be reversibly switched by varying the electric field or temperature when heating is done using a laser pulse in optical recording applications^6^,^9^,^10^. The unique characteristics of phase change GST materials are based on huge optical reflectivity (up to 30%) or electrical conductivity (several orders of magnitude) changes proceeding upon phase transition^11^,^12^.

Nowadays, commercial optical data storage media are based on mentioned phase changes. Material optimization has led to the development of three generation of optical products; apart from compact disk (CD), digital versatile disks (DVD), Blu-ray disk (BD) or high-definition digital versatile disks (HD DVD) were developed^2^. A typical material used in early optical discs was Ge_2_Sb_2_Te_5_, where the ratio (GeTe):(Sb_2_Te_3_) is 2:1. Other important composition from GST system is Ge_8_Sb_2_Te_11_, where (GeTe):(Sb_2_Te_3_) = 8:1, known as a material for the third generation optical storage (BD). The reasons for the application of Ge_8_Sb_2_Te_11_ material are fast encoding rate, high stability of the amorphous phase, and also high optical contrast between crystalline and amorphous states in the blue-violet spectral region^13^,^14^,^15^,^16^,^17^,^18^.

The structure of crystalline phases of GeTe-rich GST alloys have been studied for example by Matsunaga^17^. In most of the cases, both high-temperature cubic and low-temperature rhombohedral metastable phases exist^7^,^17^.

In the high-temperature phase, Te atoms occupy 100% of the 4(*a*) site in the space group 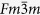, while Ge and Sb atoms are randomly located at the 4(*b*) site, which form a NaCl type cubic structure. However, an important structural feature is that the 4(*b*) site is occupied by, besides the Ge/Sb atoms, about 9 at.% intrinsic vacancies (for Ge_8_Sb_2_Te_11_)^17^.

Low-temperature mestastable phase is slightly distorted NaCl-type structure, where Te atoms occupy 100% of the (1*a*) site in the space group R3m, while Ge and Sb atoms (and vacancies) are randomly located at another (1*a*) site and Te and Ge/Sb layers in the cubic close-packed structure of the high-temperature phase are shifted away from each other in the [111] direction. The low-temperature rhombohedral phase is expected to be present from pure GeTe to near (GeTe):(Sb_2_Te_3_) = 6:1, i.e. Ge_6_Sb_2_Te_9_^17^.

The structural details of the GSTs amorphous phase are not completely understood but in recent years, significant progress has been reported^5^. Akola concluded that amorphous Ge_8_Sb_2_Te_11_ have total coordination numbers 4.0 for Ge, 3.7 for Sb, 2.9 for Te, and partial coordination number 0.7 for Ge-Ge^13^. In case of amorphous Ge_2_Sb_2_Te_5_, total coordination number were found to be Ge: 4.2, Sb: 3.7, Te: 2.9, and partial coordination number 0.4 for Ge-Ge^19^. Further, calculations show that in amorphous Ge_2_Sb_2_Te_5_, approximately 1/3 of Ge atoms are in tetrahedral geometry and remaining 2/3 of Ge atoms as well as Sb and Te atoms are in defective octahedral-like sites, similarly to cubic GST^5^,^20^. The structural differences between amorphous Ge_2_Sb_2_Te_5_ and Ge_8_Sb_2_Te_11_ seem to be small but significant: the latter has a larger Ge-Ge coordination number and a larger fraction of Ge atoms (42%) with tetrahedral coordination. On the other hand, amorphous GeTe differs from both Ge_2_Sb_2_Te_5_ and Ge_8_Sb_2_Te_11_ in its ring distribution, the increased number of Ge-Ge bonds, and much lower vacancy volume^13^.

For the fabrication of GST thin films, magnetron sputtering^1^,^21^,^22^, thermal (flash) evaporation^23^,^24^, chemical vapor deposition using metal-organic precursors^25^,^26^ or also atomic layer deposition^27^ can be used. In line with mentioned techniques, pulsed laser deposition (PLD) is a suitable deposition method for the thin films growth too. Favoritism of PLD concerns mainly its simplicity, easy process control, high deposition rate, and often stoichiometric transfer of target material to the films^28^,^29^,^30^. We already reported applicability of PLD for GST films’ fabrication^31^,^32^.

The aim of this work is to combine PLD as an advanced technique for thin films growth with the fabrication of an important class of inorganic materials being presented by GST thin films with high proportion of GeTe.

Thus, in this paper, GST thin films with different (GeTe):(Sb_2_Te_3_) ratio, specifically 6:1, 8:1, 10:1, and 12:1, i.e. Ge_6_Sb_2_Te_9_, Ge_8_Sb_2_Te_11_, Ge_10_Sb_2_Te_13_, and Ge_12_Sb_2_Te_15_ layers, were fabricated by PLD. Their characterization in as-deposited state (amorphous phase) as well as in crystalline state (induced by thermal annealing) is performed on the basis of atomic force microscopy (AFM), scanning electron microscopy (SEM) with energy-dispersive X-ray analysis (EDX), X-ray diffraction (XRD) and reflectometry (XRR), Raman scattering spectroscopy, electrical resistivity, and variable angle spectroscopic ellipsometry (VASE) data.

## Results

**Figure 1:**
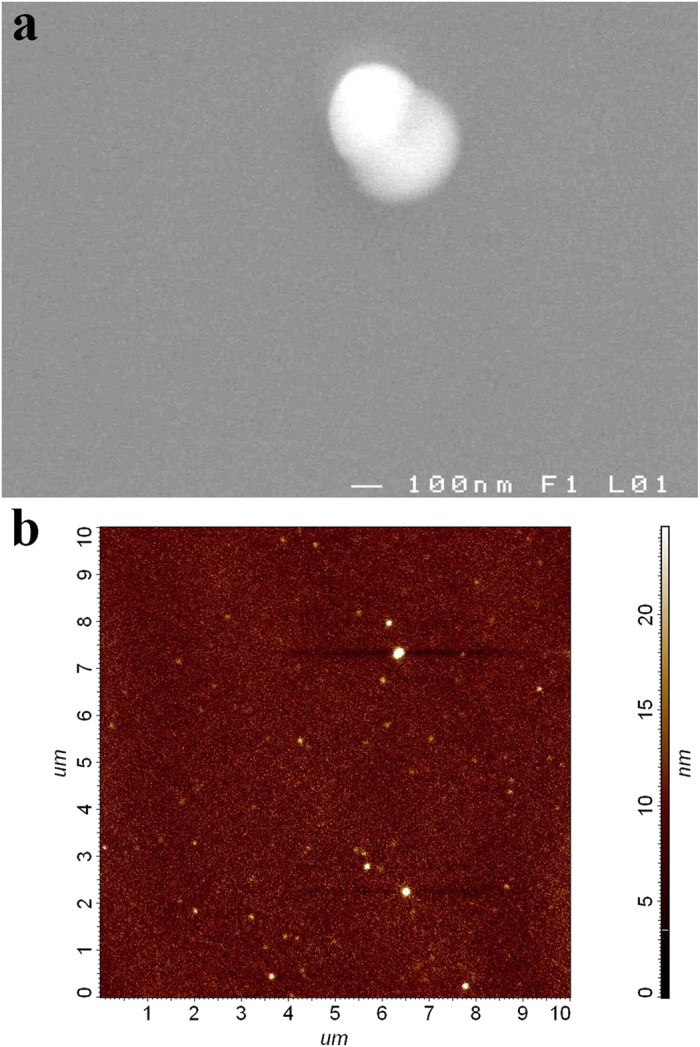
SEM micrograph (**a**) and AFM scan (**b**) of as-deposited Ge_8_Sb_2_Te_11_ thin film.

Thin films fabricated by PLD were amorphous and homogeneous. These characteristics were confirmed by optical/electron microscopy and XRD patterns. The morphology of the layers is of good quality as indicated by SEM and AFM (Fig. 1). The SEM and AFM data showed smooth surface of thin films, without cracks and corrugations. We observed only rarely sub-micrometer sized droplets (Fig. 1a). For all amorphous thin films, root mean square (RMS) roughness values determined by AFM were typically found to be lower than ~0.7 nm. On the other hand, RMS roughness determined by AFM for crystallized layers was in the range of ~0.7–0.9 nm.

The chemical composition of the films is summarized in Table 1. EDX analysis shows that in comparison with the nominal composition, the as-deposited films are slightly overstoichiometric in Ge content (4.0–5.9%). Sb content was preserved very well (overstoichiometry of 0.3–1.0%). Te deficit is reported for all the layers (−6.9 at.% at maximum). The tellurium deficiency could be caused by higher volatility of the element in comparison with Ge or Sb. We note that the error limit of the used EDX method is ±1 at.%.

**Figure 2:**
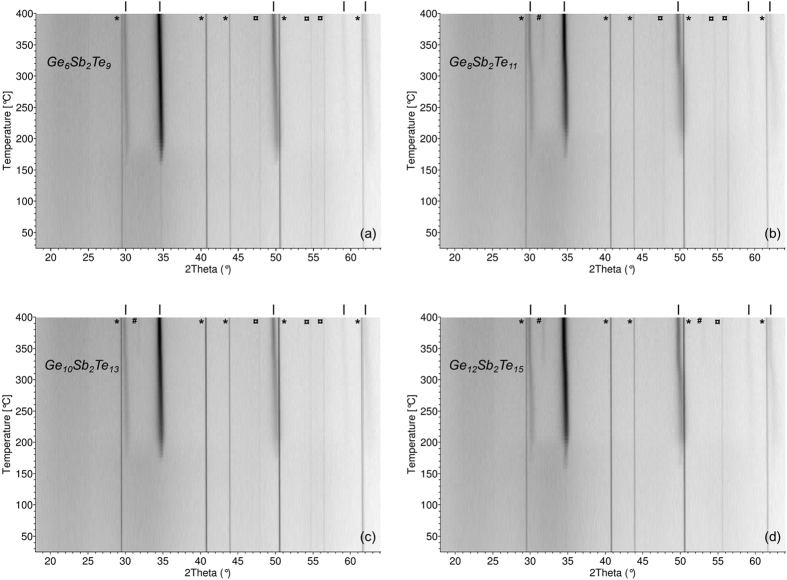
Temperature dependent XRD patterns of Ge_6_Sb_2_Te_9_ (**a**), Ge_8_Sb_2_Te_11_ (**b**), Ge_10_Sb_2_Te_13_ (**c**), and Ge_12_Sb_2_Te_15_ (**d**) PLD thin films. *Symbols are due to XRD signal coming from Al_2_O_3_ high-temperature sample cell. ^¤^Symbols describe signal of the substrate (Si). ^|^Symbols show diffraction angles of crystallized GST thin films. ^#^Denote diffraction angles of possible traces of cubic Ge.

Temperature dependent XRD data of GeTe-rich GST thin films deposited on Si substrates are shown in Fig. 2. Room temperature XRD patterns confirm amorphous state of the deposited GST layers. The initially amorphous films start to crystallize at around 165 °C and do not change approximately up to 315 °C. When indexing patterns measured at 260 °C as an example, cubic symmetry (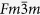) fits experimental data well. At that temperature, the XRD patterns of all four films’ compositions are quite similar; they do not show any indication for rhombohedral distortion of the cubic unit cell as it was previously reported for crystalline GST thin films^14^. This would imply the splitting of the cubic reflections (111)_C_ and (220)_C_ at 30.1° and 50.1°, respectively, into rhombohedral ones (003)_R_, (101)_R_ and (104)_R_, (110)_R_. This is not really observed in measured data. It should be noted that it is difficult to argue for a possible weak crystal structure distortion solely on the basis of XRD data measured on thin film samples. In fact, peaks in the resulting diffraction patterns are frequently rather broad (as it is the case in this work) and one cannot completely exclude the existence of weak multiple peaks splitting under the envelope of a single observed peak. However, Matsunaga *et al*.^17^ had previously worked on powdered thin film samples and had investigated the low-temperature rhombohedral to high-temperature cubic structure transition in GeTe-rich GST phases. They showed that the critical temperature of the low-temperature rhombohedral distortion of the cubic unit cell gradually decreases with increased Sb_2_Te_3_ concentration. Thus, for (GeTe):(Sb_2_Te_3_) ratios up to 6:1, i.e. Ge_6_Sb_2_Te_9_, the phases are cubic whatever the temperature is. Phases with higher ratios 8:1, 10:1, and 12:1, which are also under investigation in this work, were found to be rhombohedral below approximately 112, 147, and 190 °C, respectively. In the light of these results we could reasonably believe that when our thin films start to crystallize around 165 °C, they adopt the high-temperature cubic symmetry.

At higher temperatures, diffraction peaks of the thin films move slightly towards lower angles which is consistent with the thermal expansion of the cubic unit cell. However, above 315 °C, for three of the Ge-richest samples – Ge_8_Sb_2_Te_11_, Ge_10_Sb_2_Te_13_, and Ge_12_Sb_2_Te_15_, we can clearly see a sharp shift in the positions of the peaks at 30.1° and 50.1°. Simultaneously, in the same three samples, there is a weak additional peak starting to grow at 31.8° and a second extra one is also visible at 53.1° in Ge_12_Sb_2_Te_15_ (Fig. 2). These two additional peaks are perfectly consistent with the XRD pattern of cubic Ge. This last finding might be surprising but it could be connected with observed overstoichiometry of Ge in the deposited films (Table 1).

**Figure 3:**
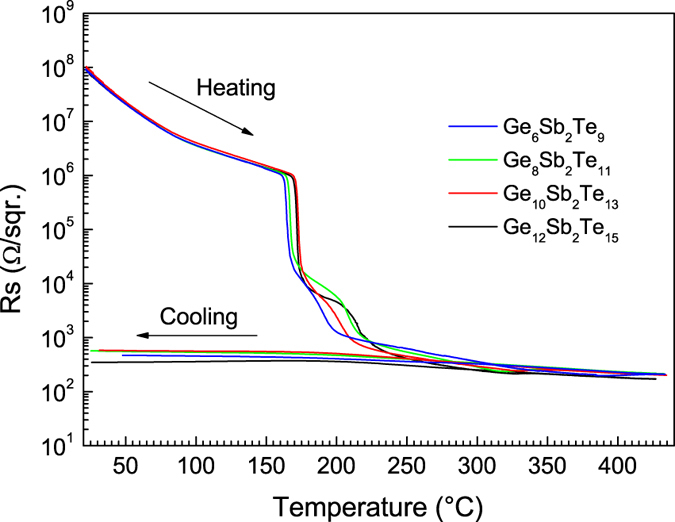
Sheet resistance of as-deposited GST layers (deposited on microscope glass substrates) on heating followed by cooling: Ge_6_Sb_2_Te_9_ (blue), Ge_8_Sb_2_Te_11_ (green), Ge_10_Sb_2_Te_13_ (red), and Ge_12_Sb_2_Te_15_ (black).

The crystallization temperatures were also evaluated on the basis of temperature dependences of thin films’ sheet resistance. The crystallization of the amorphous (GeTe)_1−x_(Sb_2_Te_3_)_x_ PLD films was identified in the region of ~164–172 °C for (Table 1) as indicated by abrupt decrease of sheet resistance (Fig. 3) and more precisely determined as peak temperatures of d(ln R_S_)/d(1/T) first derivatives. The crystallization temperatures are only slightly dependent on decreasing content of Sb_2_Te_3_ in the films and are in very good agreement with temperature dependent XRD data. Simultaneous reflectivity measurements at 650 nm show an abrupt increase of reflectivity on crystallization; it grows with increasing content of GeTe in the layers reaching saturated values of ~39–46% larger in crystalline state in comparison with the amorphous films.

**Figure 4:**
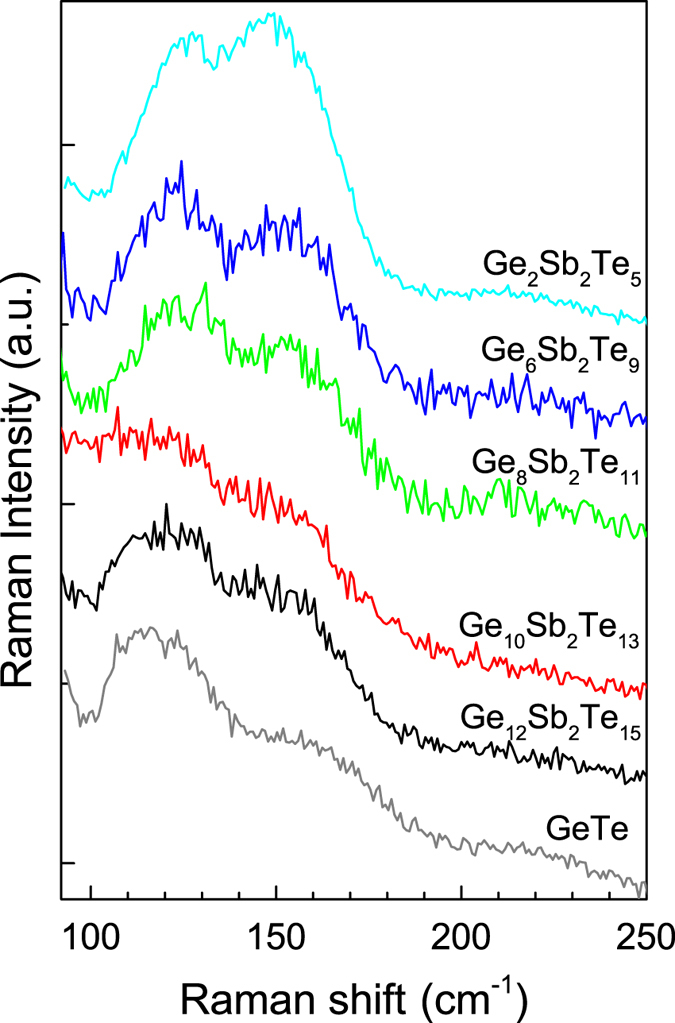
Raman scattering spectra of as-deposited amorphous PLD Ge_6_Sb_2_Te_9_ (blue), Ge_8_Sb_2_Te_11_ (green), Ge_10_Sb_2_Te_13_ (red), and Ge_12_Sb_2_Te_15_ (black) thin films. For a comparison, Raman data for amorphous GeTe (grey) and Ge_2_Sb_2_Te_5_ (cyan) PLD layers are shown.

Raman scattering spectroscopy results for studied PLD GST film in as-deposited state are presented in Fig. 4. The Raman response of all the thin films covers ~90–250 cm^−1^ region; it contains at least two bands peaking at ~120–125 and ~145–150 cm^−1^. A broad, very weak Raman band can be located at higher frequencies peaking at ~215–220 cm^−1^.

The VASE experimental data were analyzed using a three layer model of optical functions: (i) the substrate (float glass slides), (ii) the chalcogenide thin film, and (iii) the surface layer. To describe the optical response of the chalcogenide layers in the whole measured spectral region, the CL model (see Methods) was applied. For the crystallized thin films, Drude-type contribution was added to the CL model. The surface layer was defined by the effective medium approximation (thin film material mixed with 50% voids). The suitability of the CL model for the analysis of VASE data was confirmed by low values of mean square error (MSE); the maximal value of MSE = 2.81.

**Figure 5:**
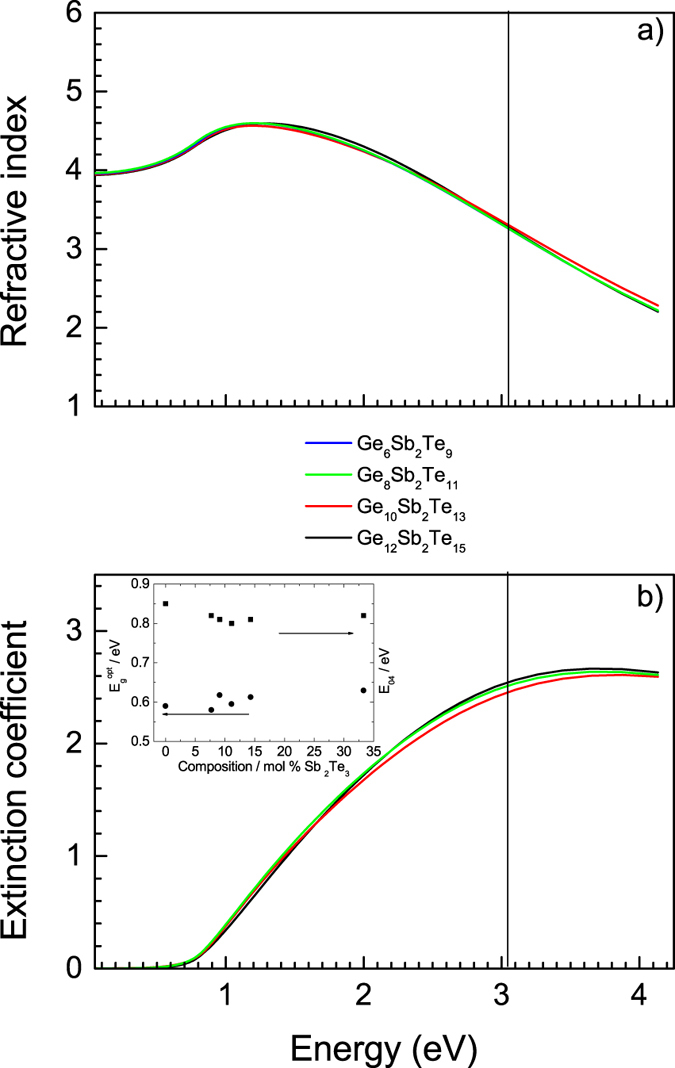
Optical functions of amorphous PLD GST thin films: (**a**) spectral dependences of refractive indices, (**b**) spectral dependences of extinction coefficients. Vertical lines represent wavelength of 405 nm used in BD technology. Inset shows optical band gap (circles) and E_04_ (squares) compositional dependences.

**Figure 6:**
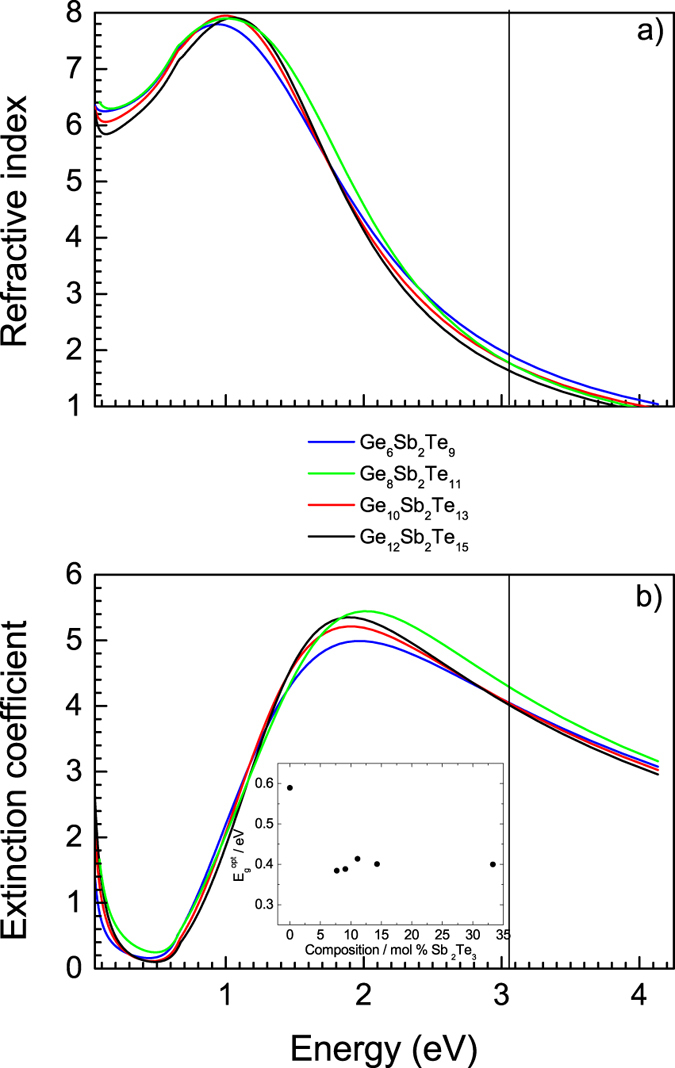
Optical functions of crystallized PLD GST thin films: (**a**) spectral dependences of refractive indices, (**b**) spectral dependences of extinction coefficients. Vertical lines represent wavelength of 405 nm used in BD technology. Inset shows optical band gap (circles) compositional dependence.

**Figure 7:**
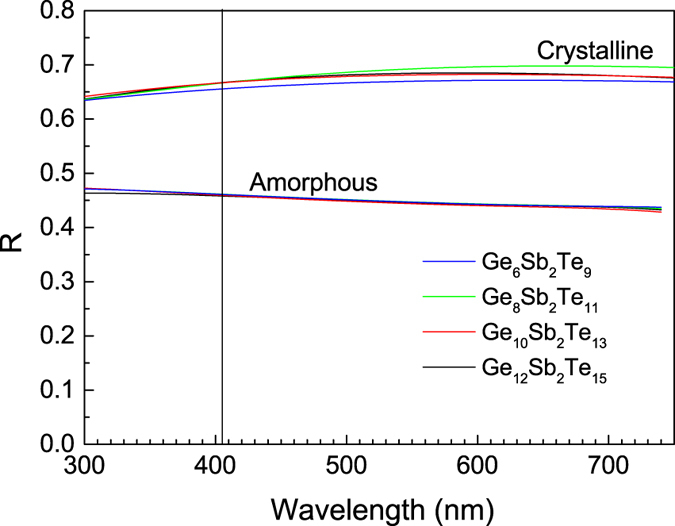
Calculated reflectivity spectral dependences for both, amorphous and crystallized PLD GST layers. Vertical line represents wavelength of 405 nm used in BD technology.

Resulting best fit optical functions, i.e. refractive index and extinction coefficient spectral dependences of amorphous as well as crystalline GST thin films are shown in Figs 5 and 6. Optical band gap values (Table 2, Figs 5 and 6) and E_04_ parameter (Fig. 5, meaning energy where the absorption coefficient *α* exceeds 10^4^ cm^−1^; *α* = *4πk/λ*, where *k* is the extinction coefficient and *λ* is the wavelength) were extracted from VASE data employing CL model. Other data resulting from the application of CL model for the analysis of VASE data (thickness ratios between crystalline and amorphous state, thickness nonuniformity values, surface roughnesses, and optical contrast defined as Δ*n* + *i*Δ*k ≈ n,k (crystalline*) *-n,k (amorphous*) at the wavelength of ~400 nm (~3.1 eV)) are summarized in Table 2. Using VASE data, reflectivity spectral dependences of both, amorphous and crystalline GST films were also calculated (Fig. 7).

Finally, the thickness ratios (crystallized/amorphous) and densities of as-deposited as well as crystallized GST thin films extracted from XRR data are given in Table 3.

## Discussion

The local structure of the as-deposited GST films was determined on the basis of Raman scattering spectroscopy data (Fig. 4). The Raman band with maxima at ~120–125 cm^−1^ can be assigned to the vibrations of GeTe_4−n_Ge_n_ (n) = 1, 2) corner-sharing tetrahedra (A_1_ mode) according to the assignment in the amorphous GeTe layers^33^. However, other (probably larger) part of the structure of GST amorphous layers can be formed by Ge-based defective octahedra according to the interpretation of Raman bands with maxima at 129 and 152 cm^−1^ based on ab initio molecular dynamics simulations performed by Caravati^20^, Mazzarello^34^, and Sosso^35^. Raman band peaking at ~145–150 cm^−1^ can also be connected partly with Sb-Te vibrations in SbTe_3_ pyramidal units^32^ or with defective octahedral coordination of Sb atoms^35^,^36^. A Raman band with maxima at ~90 cm^−1^ (not shown in Fig. 4) which could be attributed to the Γ_3_ (E) mode as in single-crystalline α-GeTe^37^ remains difficult to detect in used measurement configuration. Finally, very weak Raman band with a flat maximum near ~220 cm^−1^ (observable only for Ge_6_Sb_2_Te_9_ and Ge_8_Sb_2_Te_11_) can be connected with GeTe_4−n_Ge_n_ tetrahedra vibrations (antisymmetric stretching modes)^34^.

As it follows from Figs 5 and 6, in studied compositional region, the refractive index and extinction coefficient spectral data are not very dependent of chemical composition, especially in case of amorphous layers. In crystallized films, one can easily observe an increase of extinction coefficient for energies below ~0.4 eV, which is an indication of lower resistivity of the layers. Optical band gap values extracted from VASE data via CL model (Table 2) show independence on chemical composition; they correlate very well with data measured on GeTe (0.59 eV) and Ge_2_Sb_2_Te_5_ (0.63 eV) PLD films taking into account error limits in determination of *E*_*g*_^*opt*^ (<0.01 eV) as depicted in inset of Fig. 5. E_04_ parameter behavior follows compositional independence of *E*_*g*_^*opt*^ (inset of Fig. 5) being in very good agreement with already reported data. As an example, E_04_ of PLD Ge_8_Sb_2_Te_11_ film is found 0.80 ± 0.01 eV, which agrees well with the value of 0.82 eV published by Buller *et al*.^14^ for sputtered layer of the same composition. On crystallization, *E*_*g*_^*opt*^ values of studied GST thin films decrease of about 0.2 eV (Table 2, inset of Fig. 6) in accordance with previously reported results for Ge_2_Sb_2_Te_5_^22^,^38^.

The thickness of all the as-deposited samples under study, as determined by VASE data analysis, was in the range of ~150–200 nm. Contrary, the thickness of annealed layers was ~10–12% lower in comparison with the thicknesses of amorphous layers (Table 2). The decrease of the thickness upon crystallization of amorphous GST films is perfectly coherent with value observed for GeTe PLD films (12.5%) reported earlier^31^.

Due to the amorphous-to-crystalline phase transition, VASE surface roughness increases (Table 2). AFM results show qualitatively similar tendency, i.e. increase of surface roughness of GST films on crystallization. However, absolute values of surface roughness are larger for VASE data which is caused by the two facts: i) VASE surface roughness layer defined by effective medium approximation includes physical surface roughness as well as layer formed by native oxidation processes and ii) VASE measurement spot is much larger than measured area in case of AFM. VASE thickness nonuniformity is between 3.5 and 5.1% for as-deposited GST films, while it grows on crystallization to 9.2–12.6% (Table 2).

The values of optical contrast Δ*n + i*Δ*k* at 400 nm, which is important for Blu-ray disc technology, are presented in Table 2. For the Ge_2_Sb_2_Te_5_ and GeTe films prepared by pulsed laser deposition, optical contrast values of −1.13 + *i*0.98 and −1.98 + *i*1.45, respectively, were derived^31^. Optical contrast values reported in this work lie between mentioned values in accordance with the chemical composition of the films. It is evident that larger content of GeTe in GST layers leads to higher value of optical contrast. We note that optical contrast values calculated for Ge_6_Sb_2_Te_9_ and Ge_8_Sb_2_Te_11_ PLD thin films are in good agreement with the ones published by Yamada^6^.

Based on calculated reflectivity spectral dependences depicted in Fig. 7, the reflectivity differences between amorphous and crystalline GST films were found ~0.21 at 405 nm for Ge_8_Sb_2_Te_11_, Ge_10_Sb_2_Te_13_, and Ge_12_Sb_2_Te_15_ layers, somewhat higher in comparison with Ge_6_Sb_2_Te_9_ thin films (Δ*R *~ 0.19), proving again importance of GeTe content for this class of phase-change memory materials.

Following XRR results collected in Table 3, one can conclude that upon crystallization, the thicknesses of the films decrease of ~9–11% in excellent agreement with the values obtained using VASE (Table 2). The densities of the films increase of ~5–11% on crystallization as deducted from the densities values which are also listed in Table 3. Observed density increase agrees well with XRR data published for GST films such as Ge_2_Sb_2_Te_5_ (~6% increase)^39^, Ge_4_Sb_1_Te_5_ (~9% increase)^40^, and Ge_8_Sb_2_Te_11_ (~9% increase)^14^ prepared by DC magnetron sputtering.

## Methods

### Samples preparation

The amorphous thin films were fabricated using the polycrystalline chalcogenide targets with nominal composition of Ge_6_Sb_2_Te_9_, Ge_8_Sb_2_Te_11_, Ge_10_Sb_2_Te_13_, and Ge_12_Sb_2_Te_15_, i.e., (GeTe)_1−x_(Sb_2_Te_3_)_x_, where x = 0.14, 0.11, 0.09 and 0.077. The polycrystalline targets were prepared by the melt quenching method with high-purity elements (Ge, Sb, and Te of 5N purity) in evacuated and sealed silica ampoules; the melting was performed at a temperature of 960 °C. For the fabrication of amorphous thin films, the PLD technique was used employing a KrF excimer laser with the following parameters: wavelength of 248 nm, output energy of 300 ± 3 mJ per pulse, pulse duration of 30 ns, repetition rate of 20 Hz, energy fluence on the target ≈2.6 J cm^−2^, and the number of laser pulses 6000–7200. The laser beam was incident on the target under an angle of about 45°. Amorphous thin films were deposited in a vacuum chamber (background pressure <4.2 × 10^−4^ Pa). The substrates used for PLD (chemically cleaned microscope glass slides, Si wafers) were positioned parallel to the target surface at target to substrate distance of 5 cm. Both the target and the substrates were rotated in off-axis geometry in order to avoid damage to the target and to improve the thickness homogeneity of the films, respectively.

To study the effect of annealing, as-deposited films were annealed in inert atmosphere (pure argon) at the temperature of 300 °C for 120 min and slowly cooled down to room temperature.

### Morphological, compositional, and structural characterization

The surface of the GST films and their chemical composition were studied using scanning electron microscopy (SEM, JEOL JSM 6400) linked with energy-dispersive x-ray analyzer.

The X-Ray diffraction (XRD) experiments were performed on a PANalytical X’Pert Pro MPD diffractometer in Bragg-Brentano geometry, equipped with an Anton Paar HTK 1200 oven chamber, X’Celerator detector and using Co Kα radiation. Patterns within the 10° ≤ 2θ ≤ 75° range were collected in 47 minutes at constant temperatures, starting at room temperature and up to 400 °C with steps of 10 or 5 °C. Dried He gas atmosphere was used to prevent oxidation of the samples and a 1 °C/min heating rate was applied between steps. X-ray reflectometry (XRR) measurements were performed at room temperature on the as-deposited thin films, then on the annealed samples after the high-temperature XRD. A Bruker D8 Discover diffractometer with a horizontal goniometer and a ¼-circle Eulerian cradle was used - Cu Kα radiation, Göbel mirror and LynxEye detector in a 0D-mode.

Atomic force microscopy (Solver NEXT, NT-MDT) was used to study topography of Ge-Sb-Te thin films within typical scanned area 10 μm × 10 μm (or 5 μm × 5 μm) in semicontact mode.

Raman scattering spectra were measured at room-temperature by double-monochromator Raman spectrophotometer HR800 (Horiba Jobin-Yvon) with 785 nm laser diode as excitation source for 600 sec averaging 4 accumulations. Light intensity of laser beam on the sample was kept at low level (~0.13 mW.μm^−2^) to avoid changes of Raman spectra due to thermally-induced structural transformations induced by absorption of high laser power densities. The wavenumber resolution of used configuration was ~0.7 cm^−1^ per pixel. For used measurement configuration, the low energy cut-off of the Raman data is around 70 cm^−1^; however, one can get reasonable data from ~90 cm^−1^.

### Electrical and optical characterization

Simultaneous optical reflectivity (R) and sheet resistance (R_S_) temperature dependences measurements (at the heating rate of 2 °C min^−1^) were realized with a homemade setup employing 650 nm laser diode and using aluminum layer reflectivity as a standard (R_Al_~100%). Sheet resistance measurements were carried out using a four-point probe based on van der Pauw method^41^.

Two variable angle spectroscopic ellipsometers (J. A. Woollam Co., Inc., Lincoln, NE, USA) were employed for the optical characterization of studied materials: first ellipsometer with automatic rotating analyzer for the spectral range 300 nm–2.3 μm (UV-NIR), measuring 100 revolutions with wavelengths steps of 20 nm at selected angles of incidence (50°, 60°, and 70°); second ellipsometer with rotating compensator for 1.7–20 μm range using angles of incidence as above, 50 scans, 15 spectra per revolution, resolution of 16 cm^−1^. For the analysis of VASE data in broad measured spectral region (300 nm–20 μm), we used Cody-Lorentz (CL) model which includes the correct band edge function, weak Urbach absorption tail description as well as Lorentz oscillator function^42^; this model is appropriate for the description of amorphous chalcogenides optical functions^31^,^43^. Drude-type contribution for free carriers was added to the CL model for crystallized samples.

## Conclusions

(GeTe)_1−x_(Sb_2_Te_3_)_x_ amorphous thin films with high content of GeTe (Ge_6_Sb_2_Te_9_, Ge_8_Sb_2_Te_11_, Ge_10_Sb_2_Te_13_, and Ge_12_Sb_2_Te_15_) were fabricated by the UV pulsed laser deposition. The quality of the prepared layers is good, as results from SEM and AFM observations. On annealing, the amorphous films undergo phase change to crystalline layers with cubic symmetry (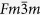) as results from temperature dependent XRD patterns. Sheet resistance measurements show that the crystallization temperatures are only slightly dependent on increasing content of GeTe. Optical functions and electrical resistance of the films change drastically upon crystallization of the films, leading to large optical (up to ~0.21 at 405 nm reflectivity contrast) and electrical (almost 6 orders of magnitude sheet resistance decrease) differences between amorphous and crystalline phases. Optical band gap values (~0.6 and ~0.4 eV for amorphous and crystalline films, respectively) are almost independent of GeTe content. Increase of thicknesses, densities, and roughnesses of the layers arising due to the crystallization accompany huge changes of optical and electrical properties. As results from Raman scattering spectroscopy data, local structure of the amorphous GST films is formed by GeTe_4−n_Ge_n_ (n) = 1, 2) tetrahedra, Ge-based defective octahedra, SbTe_3_ pyramids and/or defective octahedral coordination of Sb atoms. All the obtained data confirm the importance of GeTe content in (GeTe)_1−x_(Sb_2_Te_3_)_x_ thin films and only marginal changes of films’ physicochemical properties below x < 0.14.

## Additional Information

**How to cite this article**: Bouška, M. *et al*. Pulsed laser deposited GeTe-rich GeTe-Sb_2_Te_3_ thin films. *Sci. Rep.* **6**, 26552; doi: 10.1038/srep26552 (2016).

## Figures and Tables

**Table 1:** Basic characteristics of PLD GST films: nominal and real chemical composition (from EDX, ±1 at.%) and crystallization temperatures (*T*_*c*_) determined from sheet resistance measurements (±1 °C).

**Nominal composition (at.%)**	**Real composition (at.%)**	**T**_**c**_ **(°C)**
Ge_35.3_Sb_11.8_Te_53.0_ (Ge_6_Sb_2_Te_9_)	Ge_39.9_Sb_12.1_Te_48.0_	163.5
Ge_38.1_Sb_9.5_Te_52.4_ (Ge_8_Sb_2_Te_11_)	Ge_44.0_Sb_10.5_Te_45.5_	166.4
Ge_40.0_Sb_8.0_Te_52.0_ (Ge_10_Sb_2_Te_13_)	Ge_44.0_Sb_8.9_Te_47.1_	171.7
Ge_41.4_Sb_6.9_Te_51.7_ (Ge_12_Sb_2_Te_15_)	Ge_46.6_Sb_7.8_Te_45.6_	170.6

**Table 2:** PLD GST films optical band gap (*E*_*g*_^*opt*^, ±0.01 eV), optical contrast Δ*n*+*i*Δ*k (n,k (crystalline)-n,k (amorphous*)) values at wavelength of 400 nm, thickness (*d*) ratios between crystalline and amorphous state, thickness nonuniformity values (*TNU*), surface roughnesses (*SR*), in both amorphous and crystalline state.

**Nominal composition**	***E***_***g***_^***opt***^ **(am.) (eV)**	***E***_***g***_^***opt***^ **(cr.)(eV)**	**d(cr.)/d(am.) VASE**	***TNU*** **(am.) (%)**	***TNU*** **(cr.) (%)**	***SR*** **(am.) (nm)**	***SR*** **(cr.) (nm)**	**Δ*****n***** + *****i*****Δ*****k***
Ge_6_Sb_2_Te_9_	0.61	0.40	0.887	3.5 ± 0.1	12.6 ± 0.3	2.4 ± 0.11	4.7 ± 0.06	−1.34 + *i*1.47
Ge_8_Sb_2_Te_11_	0.60	0.41	0.893	3.9 ± 0.2	12.3 ± 0.3	2.3 ± 0.11	6.1 ± 0.07	−1.49 + *i*1.70
Ge_10_Sb_2_Te_13_	0.61	0.39	0.884	5.1 ± 0.1	10.1 ± 0.1	1.5 ± 0.08	5.2 ± 0.06	−1.53 + *i*1.51
Ge_12_Sb_2_Te_15_	0.58	0.38	0.901	4.1 ± 0.2	9.2 ± 0.2	4.4 ± 0.09	6.8 ± 0.08	−1.64 + *i*1.40

Error in determination of thickness is ±2 nm. All data are extracted from VASE data analysis via CL model.

**Table 3:** Thickness (*d*) ratios between crystalline and amorphous state and densities (±0.1 g.cm^−3^) of amorphous and crystalline GST thin films extracted from XRR data.

**Nominal composition**	**d(cr.)/d(am.) XRR**	**density (am.) (g.cm**^**−3**^)	**density (cr.) (g.cm**^**−3**^)
Ge_6_Sb_2_Te_9_	0.887	5.8	6.2
Ge_8_Sb_2_Te_11_	0.908	5.6	6.2
Ge_10_Sb_2_Te_13_	0.904	5.8	6.1
Ge_12_Sb_2_Te_15_	0.891	5.8	6.2

Error in determination of thickness is ±4 nm.
